# Does positive MGMT methylation outbalance the limitation of subtotal resection in glioblastoma IDH-wildtype patients?

**DOI:** 10.1007/s11060-021-03794-8

**Published:** 2021-06-29

**Authors:** Müller Mareike, Staub-Bartelt Franziska, Ehrmann Julia, Hänggi Daniel, Sabel Michael, Felsberg Jörg, Rapp Marion

**Affiliations:** 1 Department of Neurosurgery, University Hospital Duesseldorf, Duesseldorf, Germany; 2 Department of Neuropathology, University Hospital Duesseldorf, Duesseldorf, Germany; 3 Department of Neurosurgery, Medical Faculty, Heinrich-Heine University Düsseldorf, Moorenstraße 5, 40225 Düsseldorf, Germany

**Keywords:** Glioblastoma, Neurooncology, MGMT, Extend of resection, Subtotal resection

## Abstract

**Background:**

The impact on survival of complete resection (CR) in patients with malignant glioma and MGMT promoter methylation on adjuvant therapy strategies has been proven in the past. However, it is not known whether a MGMT promoter methylation can compensate a subtotal resection. Therefore, we analyzed the progress of postoperative residual tumor tissue depending on the molecular tumor status.

**Methods:**

We included all glioblastoma, IDH-wildtype (WHO grade IV) patients with postoperative residual tumor tissue, who were treated at our neurooncological department between 2010 and 2018. Correlation of molecular patterns with clinical data and survival times was performed. The results were compared to patients following CR.

**Results:**

267 patients with glioblastoma, IDH-wildtype (WHO grade IV) received surgery of whom 81 patients with residual tumor were included in the analysis. MGMT promoter was methylated in 31 patients (38.27%). Median OS and PFS were significantly increased in patients with methylated MGMT promoter (mOS: 16 M vs. 13 M, p = 0.009; mPFS: 13 M vs. 5 M, p = 0.003). In comparison to survival of patients following CR, OS was decreased in patients with residual tumor regardless MGMT methylation.

**Conclusion:**

Our data confirm impact of MGMT promoter methylation in patients with glioblastoma, IDH-wildtype on OS and PFS. However, in comparison to patients after CR, a methylated MGMT promoter cannot compensate the disadvantage due to residual tumor volume. In terms of personalized medicine and quality of life as major goal in oncology, neuro-oncologists have to thoroughly discuss advantages and disadvantages of residual tumor volume versus possible neurological deficits in CR.

## Introduction

Malignant gliomas are heterogeneous, infiltrative growing tumors and represent the most frequent diagnosed brain tumors in adults with an incidence of 5–6 per 100,000 inhabitants per year. The most common subgroup of malignant glioma is glioblastoma multiforme (GBM) mounting up to more than 50% of the malignant gliomas [1, 2]. Besides histomorphological aspects, impact of molecular-genetic tumor markers regarding diagnosis, prognosis and therapy decisions have been demonstrated [3, 4]. The use of “integrated” phenotypic and genotypic parameters for CNS tumor classification has led to the fact that glioblastomas are divided in the 2016 CNS WHO into glioblastoma, IDH-wildtype (about 90% of cases), which corresponds most frequently with the clinically defined primary or de novo glioblastoma and predominates in patients over 55 years of age [5].

As predominant markers isocitrate dehydrogenase (IDH) mutation status and O-6-methylguanine-DNA methyltransferase (MGMT) methylation have been discussed in multiple studies. IDH1/2 mutation has a positive prognostic influence on overall survival (OS) (31 months vs. 15 months) [6, 7]. Furthermore, MGMT promoter methylation was implemented in diagnostic and therapeutical considerations as studies have proven that a methylated MGMT promoter (i.e. a hypermethylation of MGMT) results in a significantly better treatment response to the standard alkylating chemotherapy with Temozolomid (TMZ). The median OS was 21.7 months in comparison to 12.7 months in patients without MGMT promoter methylation [8, 9].

Despite improvement of therapy regimes, tumor recurrence cannot be prevented. With a mean progression free survival (PFS) of 6.9 months mainly local tumor recurrence is inevitable [10].

Therefore, the actual primary gold standard to increase survival is a complete resection (CR) of the contrast enhancing tumor tissue. Even beyond whenever possible [11], respecting location, functional restrictions due to possible risks of permanent neurological deficits [12] as well as general health condition [13]. Vice versa it has been shown, that postoperative residual tumor tissue results in a decreased OS with an inverse correlation of tumor volume and survival [14, 15]. Additionally, adjuvant therapies with concomitant radio-chemotherapy according to the EORTC/NCIC 26,981–22,981 study [16] as well as additional administration of Lomustin in patients with methylated MGMT promoter, have proved to significantly increase OS [17].

To improve surgical outcome, intraoperative neuromonitoring, awake surgery or fluorescence guided surgery have become widely used standard tools in glioma surgery. Although CR or even supramarginal resection has been proven to be the gold standard for primary therapy, this is not always achievable. Neurosurgeons have to identify intraoperative limits in order to prevent permanent neurological deficits especially in eloquent located tumors. One of the most difficult and also ethical decisions is to outbalance the risk of permanent neurologic deficits, which may result in decreased health related quality of life versus leaving active tumor tissue behind, which inevitably results in decreased survival. But it is not known, if a favorable molecular tumor status may outbalance postoperative residual tumor tissue. Do we, as neurosurgeons, have to be as aggressive in MGMT positive methylated than in negative methylated tumors?

Therefore, to facilitate the decision process of “how far can you go” vs “how far must we go”, we analyzed whether a methylated MGMT promoter is able to balance the disadvantage of post-op residual tumor regarding tumor progression, PFS, OS as well as clinical outcome in IDH-wildtype glioblastoma patients. In a second step, we correlated our data with patients with no residual tumor tissue in the post-operative MRI.

## Patients and methods

In this retrospective single-center analysis we investigated the impact of MGMT status on survival of GBM patients with postoperative residual tumor. The study was approved by the local ethical committee (study number: 5632 and 3005). Reporting of this study was according to the strengthening the reporting of observational studies in epidemiology (STROBE) guidelines for observational studies (supplementary material).

### Patients

Inclusion criteria were: (1) initial diagnosis of IDH-wildtype glioblastoma between January 2010 and December 2018, (2) surgery at the neurosurgical department, University Hospital Duesseldorf, (3) availability of pre- and postoperative (< 72 h post-op) MRI scans, (4) residual tumor tissue in the early postoperative MRI, (5) neuropathological diagnosis according to the 2016 guideline, (6) availability of molecular-genetic testing of MGMT status, (7) adjuvant therapy with concomitant radio-chemotherapy followed by intermittent TMZ according to the EORTC/NCIC CTG 26,981–22,981 study.

### Molecular analyses

Since 2005 MGMT promoter analysis is conducted standardly in regards to glioma diagnostic at the Institute of Neuropathology at the University Hospital Duesseldorf. MGMT promoter methylationstatus was determined by methylation-specific PCR (MSP) and IDH mutation status was determined by immunohistochemistry for IDHR132H and DNA pyrosequencing as reported before [18].

If histopathological and molecular genetic diagnosis were provided before introduction of WHO 2016 classification, all diagnoses were reclassified according to WHO 2016 classification by the Institute of Neuropathology prior to our analysis.

### Clinical status

The Karnofsky performance status scale (KPS) was determined pre- and post-operatively in order to evaluate the clinical status of the patients during the course of disease. It was also evaluated when a recurrent or progressing tumor was diagnosed. We defined clinical deterioration as a decrease in the Karnofsky performance status scale of at least 10%. A decrease in the KPS postoperatively can be caused by a new neurologic deficit as e.g. a hemiplegia but also by other factors such as side-effects from anesthesia, thrombosis, or infection.

### Calculation of tumor volume and treatment response

Initial tumor volume was calculated at baseline (preoperative MRI) and at T0 (> 72 h postoperatively) by defining the largest diameter of contrast-enhancing tissue in three spatial dimensions and then subsequently using the simplified formula for ellipsoids (A × B × C/2) for calculating the volume [19]. When second look surgery was performed, the MRI after second look was used for calculation.

Residual tumor volume was defined as minimum tumor volume greater than or equal to 0.175 cm^3^. In further analyses we differentiated between residual tumor volume smaller or greater than 1.5 cm^3^. Follow-Up MRIs were performed every three months starting 6 weeks after completion of concomitant radio chemotherapy (T1, T2 …).

Treatment response was assessed regarding RANO criteria [20]. For evaluation of complete (CR) or partial remission (PR) current MRIs were compared to baseline MRIs. If current MRI demonstrated either CR or PR, an additional MRI after 4 weeks was performed to verify treatment response. If there was no further MRI stable disease (SD) only was defined.

According to Wen and colleagues [20] follow-up MRI with the smallest measurable residual tumor volume was used for diagnosing tumor recurrence.

Furthermore, tumor progression was defined in case of:

1.  > 25% increase of contrast-enhancement tissue in T1 + contrast with stable or increasing need for corticosteroids according to RANO criteria
2. evidence of an increasing metabolic activity of tumor tissue in the ^18^F-FET-PET examination (delimitation of progression vs. pseudoprogression)
3. re-resection with histopathological diagnosis of recurrent tumor

### Statistical analysis

Testing for normal distribution of the data was performed by using the Shapiro–Wilk test, variance homogeneity and sphericity were tested by Levene and Mauchly test. Non-parametric testing for independent samples was conducted using the Mann–Whitney *U* test and Kruskal–Wallis test. For significance testing of two categorical variables Fisher’s exact test was used.

Survival was calculated by means of the Kaplan–Meier method. In case of multivariable analysis Cox regression was used.

All statistical analysis was performed by using IBM SPSS Statistics Version 26 (IBM Corporation, USA).

OS in month was defined by the period from histopathological diagnosis to the time of death. In case of missing date of death, the last follow-up was defined as time of death. The period from diagnosis until occurrence of de novo tumor growth corresponded to PFS.

The cohorts’ survival data (median OS, median PFS) were additionally compared to data from cohorts reported with complete resection [21–23].

## Results

### Patients

**Fig. 1:**
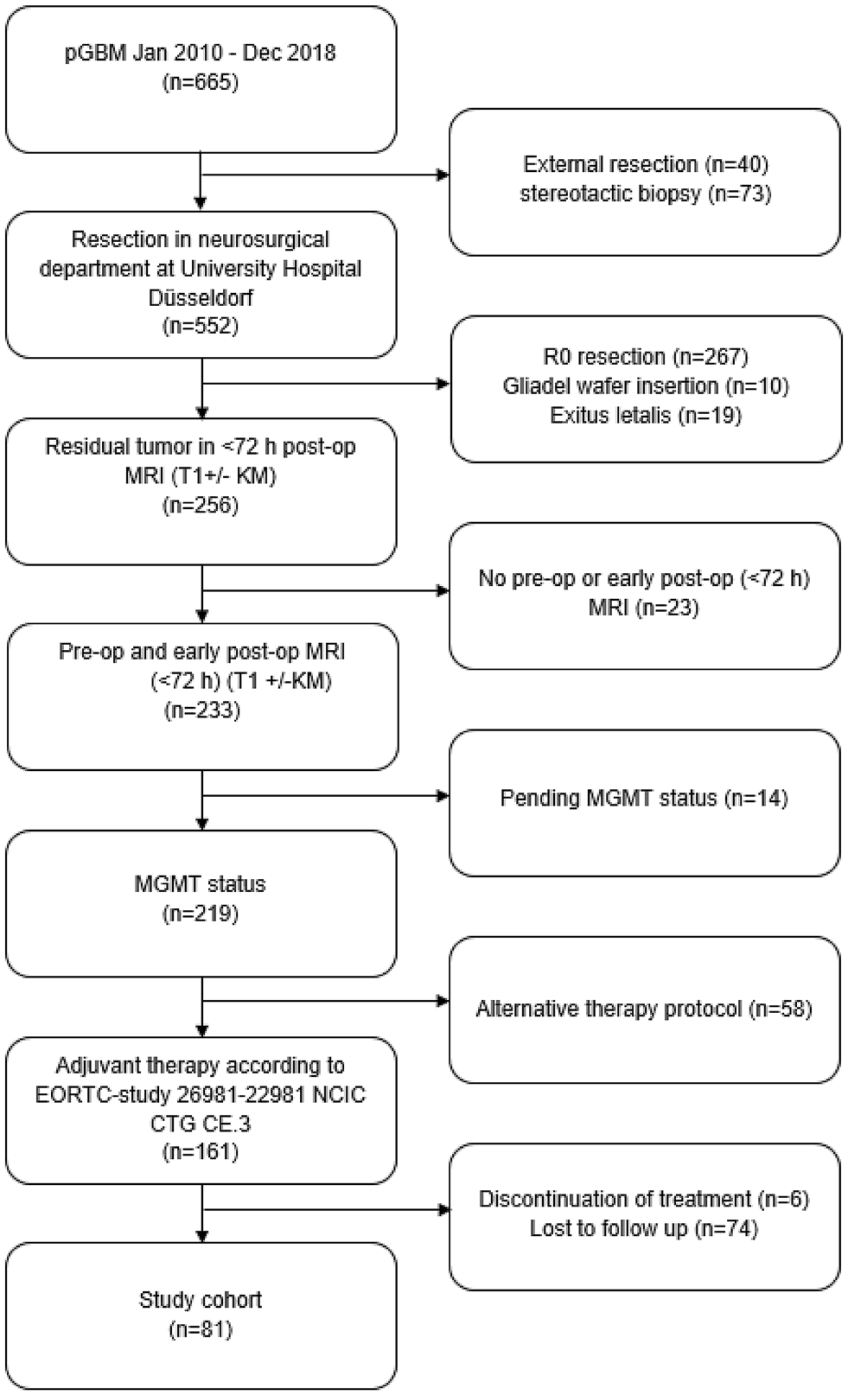
Patients’ inclusion and exclusion procedure illustrated as flow chart

Six hundred and sixty five patients with IDH-wildtype glioblastoma were treated at the neurosurgical department at the University Hospital in Duesseldorf from January 2010 until December 2018. 267 (40.15%) patients received a complete resection, in 256 (53.53%) patients, residual tumor on the early post-op MRI was diagnosed. Due to the retrospective study design and chosen inclusion criteria, finally 81 patients (male: n = 50, 61.7%) were included in the following analysis. Figure 1 illustrates the patient recruitment.

The median age at diagnosis was 63 years (range 30–86 years). The median observation period was 13 months (95% CI, 11.75–14.26). The median PFS was 7 months (95% CI, 4.97–9.04). At time of data evaluation, 77 patients (95.1%) had died. An unmethylated MGMT promoter was diagnosed in 49 (64%), a positive MGMT status in 28 (36%) tumors.

Pre-op, the KPS was > 90% in two thirds of the patients (66.7%) with a median of 90%. The median KPS did not change post-op. At the event of a local tumor progression, 37 patients (81.1%) had no change or a decline of 10% in their KPS, whereas nine patients (15.5%) had a KPS decrease from 20–30%, and two patients (3.4%) from 50%.

The tumors were located in both hemispheres equally (left n = 37, 45.7%; right n = 39, 48.2%; both n = 4, 4.9%; cerebellar n = 1, 1.2%). The most common location was the frontal lobe (35.0%), followed by the temporal lobe (22.5%). The location was considered eloquent in 92.6% leading to awake brain surgery in 43 patients (57.3%) and the application of intraoperative monitoring in 75 patients (92.6%). Incomplete resection was due to eloquent tumor location or vascular conflicts.

The median pre-op tumor volume was 36.85 cm^3^ (range 3.34–127.05cm^3^). The group of MGMT promoter methylated tumors had shown a higher median pre-op volume (43.5cm^3^) compared to the group of MGMT promoter unmethylated tumors (32.16cm^3^). Patient characteristics are illustrated in Table 1.

**Table 1:** Patient characteristics

Parameter	Value	Whole population	MGMT positive	MGMT negative
		n = 81	n = 31	n = 50
At data evaluation	Alive	4 (4.9%)	3 (9.7%)	1 (2.0%)
	Dead	77 (95.1%)	28 (90.3%)	49 (98.0%)
Sex	Male	50 (61.7%)	18 (58.1%)	32 (64.0%)
	Female	31 (38.3%)	13 (41.9%)	18 (36.0%)
Age at initial diagnosis (in years)	< 60	38 (46.9%)	13 (41.9%)	25 (50.0%)
	> 60	43 (53.1%)	18 (58.1%)	25 (50.0%)
	Median (range)	63 (30–86)	64 (46–86)	61 (30–82)
	Mean (SD)	61.74 (11.53)	63.94 (9.93)	60.38 (12.32)
KPS at initial diagnosis	60	1 (1.2%)	1 (3.2%)	0 (0%)
	70	4 (4.9%)	3 (9.7%)	1 (2.0%)
	80	9 (11.2%)	2 (6.5%)	7 (14.0%)
	90	54 (66.7%)	19 (61.2%)	35 (70.0%)
	100	13 (16.0%)	6 (19.4%)	7 (14.0%)
	Median (range)	90 (60–100)	90 (60–100)	90 (70–100)
KPS post-op	60	0 (0%)	0 (0%)	0 (0%)
	70	4 (4.9%)	2 (6.5%)	2 (4.0%)
	80	8 (9.9%)	2 (6.5%)	6 (12.0%)
	90	45 (55.6%)	15 (48.4%)	30 (60.0%)
	100	24 (29.6%)	12 (38.6%)	12 (24.0%)
	Meadian (range)	90 (70–100)	90 (70–100)	90 (70–100)

The table shows the patient characteristics of the whole population and depending on the MGMT status

### Treatment response

At the time of data evaluation, PD was diagnosed in 58 patients. 23 patients showed a treatment response (TR). There was a significant correlation between methylated MGMT promoter and therapy response (p = 0.001). Tumors with methylated MGMT promoter demonstrated a significant higher TR (51.6%) compared to the tumors with unmethylated MGMT promoter (14.0%) (Table 2).

**Table 2:** Course of disease

	Cohort n = 81 (%)	MGMT positive n = 31 (%)	MGMT negative n = 50 (%)
Treatment response (TR) n = 23			
Complete remission (CR)	2 (8.7)	2 (12.5)	0 (0)
Partial remission (PR)	2 (8.7)	1 (6.3)	1 (14.3)
Stable disease (SD)	19 (82.6)	13 (81.2)	6 (85.7)
Progressive disease (PD) n = 58			
RANO-criteria	24 (41.4)	5 (33.3)	19 (44.2)
^18^F-FET-PET	9 (15.5)	9 (6.7)	8 (18.6)
Surgery	25 (43.1)	25 (43.1)	16 (37.2)

Treatment response dependent from MGMT methylation status, and diagnosis of progressive disease

PD was diagnosed via RANO criteria in 24, via ^18^F-FET-PET in 9, and via surgery in 25 patients. In case of recurrent tumor surgery, MGMT methylation status could be verified in all patients and was identical in 100% to the MGMT status of the tissue resected in the primary surgery.

### Survival data

**Fig. 2:**
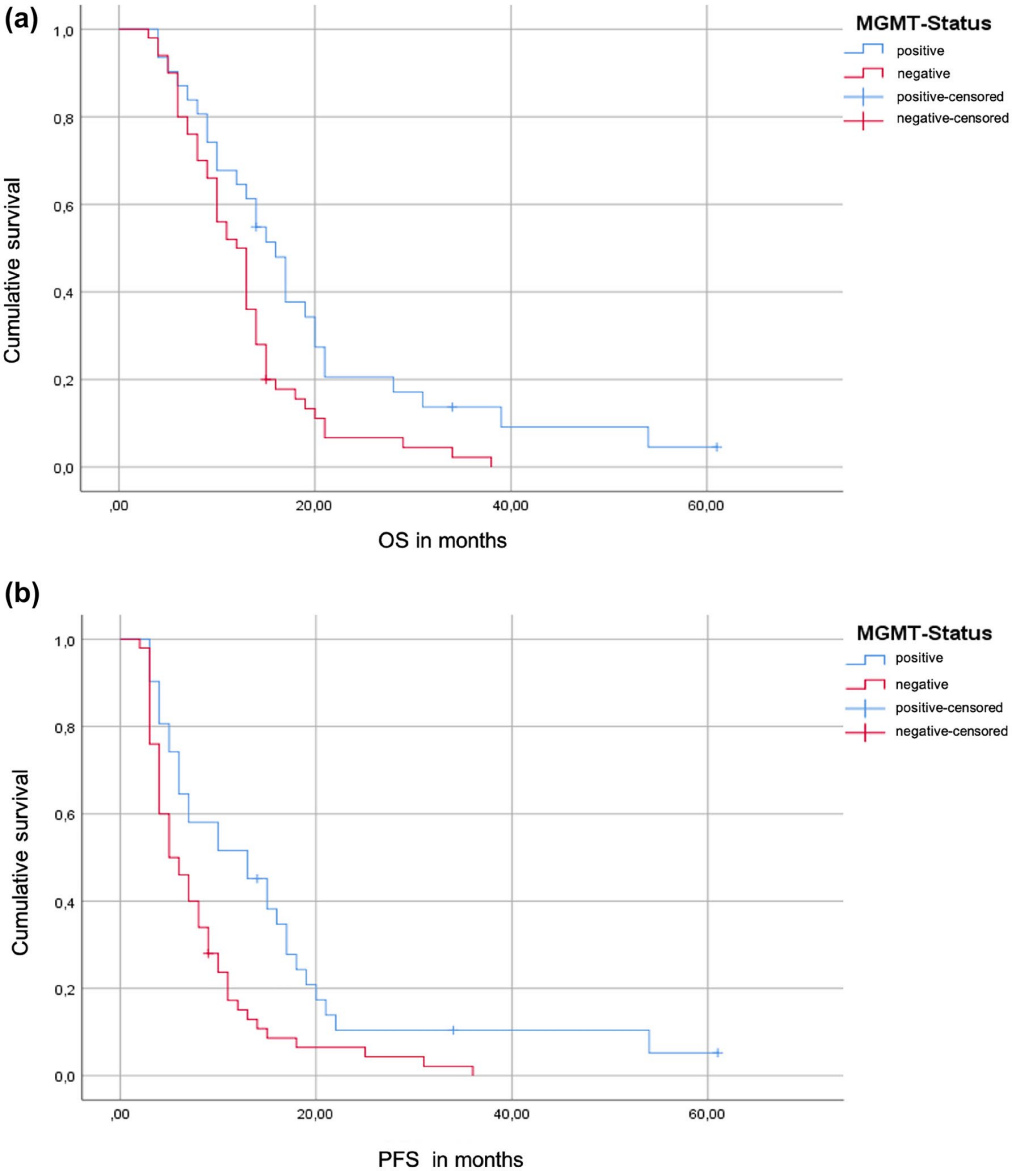
Kaplan–Meier Curve overall survival (**A**) and progression free survival (**B**)

In patients with MGMT methylated tumors a significant increased OS as well as PFS were observed (p = 0.009, p = 0.003, respectively). OS and PFS (Fig. 2A, B) in patients with MGMT hypermethylated residual tumors were 16 months (95% CI, 13.00–19.01) and 13 months (95% CI, 5.94–20.06). In MGMT negative tumors mOS was 12 months (95% CI, 9.92–14.08) and mPFS 5 months (95% CI: 2.92–7.08).

Survival data were additionally correlated to a patient cohort who received a gross total resection at the same department [22]. Comparing available data of both groups tumors mostly were located eloquently (67% vs. 92.6%). While complete resected tumors were mainly localized on the left hemisphere (58.8%), incomplete resected tumors occurred mainly on the right hemisphere (48.2%). With a median preoperative tumor volume of 36.85 cm^3 ^incomplete resected patients showed a larger preoperative tumor volume (36.85 vs. 23 cm^3^). The median preoperative KPS was 90% in both populations. Median age of patients with total resection was 58 years. An incomplete resection was associated with a higher age at time of diagnosis (median 63 years).

In this analysis, patients with a positive MGMT methylation status also demonstrated an increased OS (mOS: 21.0 vs. 19.0 months). The mPFS was 9.0 months in both groups. Compared to our data, following incomplete resection there is a similar mPFS, but increased mOS.

### Tumor progression

Independent from molecular analysis, median residual tumor volume of all patients was 0.94 cm^3^ (range: 0.18–11.50 cm^3^, Table 3). There was no significant difference regarding residual tumor tissue and MGMT methylation status (Mann–Whitney *U* test: p = 0.392). Due to the significant difference of OS and PFS in patients with MGMT positive and negative methylated tumors, tumor volume was calculated at 3, 6, and 9 months post-op (Table 4).

**Table 3:** Post-OP residual tumor volume

Value	Cohort n = 81	MGMT positive n = 31	MGMT negative n = 50
V_Rest_ in cm^3^			
≤ 1.5 cm^3^ (%)	54 (66.7)	20 (64.5)	34 (68.0)
> 1.5 cm^3^ (%)	27 (33.3)	11 (35.5)	16 (32.0)
Median (Range)	0.94 (0.18–11.50)	0.94 (0.18–8.92)	0.94 (0.18–11.50)
Mean (SD)	1.63 (2.02)	1.68 (1.88)	1.61 (2.12)

The table shows the post-op residual tumor volume (at T0 = post-op) assorted by the MGMT status

**Table 4:** Residual tumor tissue at different timepoints

Value	T0	T3	T6	T9
V_Rest_ MGMT positive in cm^3^				
n	31	26	20	13
Median (Range)	0.94 (0.18–8.92)	0.98 (0.17–10.4)	1.23 (0.21–10.75)	0.41 (0.18–5.60)
Mean (SD)	1.68 (1.88)	3.93 (10.4)	2.04 (2.58)	1.29 (1.6)
V_Rest_ MGMT negative in cm^3^				
n	49	34	12	13
Median (Range)	0.94 (0.18–11.50)	1.43 (0.2–19.36)	1.75 (0.34–30.45)	1.23 (0.2–53.7)
Mean (SD)	1.61 (2.12)	3.95 (4.99)	4.4 (8.33)	6.38 (14.87)

The table shows the course of the residual tumor tissue in patients with a MGMT positive and MGMT negative GBM at different time points: T0 = post-op, after 3 (T3), 6 (T6), and 9 (T9) months post-op

### Impact of age, residual tumor volume and KPS on survival

Our data demonstrate a negative impact of age and residual tumor volume on survival. In patients older than 60 years median OS (95% CI: 6.19–13.85) and median PFS (95% CI: 5.19–8.81) was decreased compared to younger patients (95% CI: 12.66–15.34 and 3.47–12.53). Patients with a residual tumor volume greater than 1.5 cm^3^ had a shorter median OS (95% CI: 9.00–11.00) and median PFS (95% CI: 3.09–6.91) than patients with a smaller residual tumor volume (95% CI: 3.92–10.08 and 11.89–14.11). However, these findings were not statistically significant. The pre- and postoperative analysis of the KPS could not show any significant influence on OS or PFS in our cohort.

## Discussion

In this study, we intended to analyze whether a methylated MGMT promoter could outweigh the burden of incomplete resection in IDH-wildtype glioblastoma patients. During the last years, the identification of molecular markers has been of high importance in the treatment of malignant gliomas [5, 24]. The methylated MGMT promoter is attributed to a better therapy response to adjuvant chemoradiotherapy, and hence to a better OS [3, 25, 26].

Our data support these findings, since patients with a hypermethylated MGMT promoter demonstrated an increased mOS (16 months with a mPFS of 13 months) compared to patients with unmethylated MGMT promoter residual tumors (mOS of 12 months with a mPFS of 5 motnhs (p = 0.009; p = 0.003)). On average, tumor progression in MGMT promoter methylated residual tumors was diagnosed 0.41 months later than patients with an unmethylated MGMT promoter. A possible reason for this short time difference might be the gray zone of glioblastoma with borderline methylation of the promoter region and the selection of the cut-off to which category the tumors are classified [27].

MGMT tumor methylation was also positively correlated to increased treatment response (51.6% vs. 14.0%), which is in line with the recent literature [23, 25, 26, 28, 29].

However, compared to patient cohorts following CR, our study population showed decreased survival times. Thus, our data cannot support that a preferable MGMT methylation could compensate the loss in survival times from incomplete resection.

### Surgical treatment strategies

The extent of resection is considered crucial for the further course of the disease in IDH-wildtype glioblastoma. Recently, Molinaro et al. discussed, that IDH -wild-type and IDH -mutant glioblastoma patients benefit from a maximized percentage of resection of contrast- and non-contrast enhancing tumor regardless the MGMT status [30]. Since residual tumor tissue is attributed to a reduced OS, aggressive gross total resection of at least all contrast-enhancing tumor on MRI or even beyond is the gold standard [13, 31–35]. Several surgical adjuncts can help to optimize the extent of resection without causing permanent post-op neurological deficits. They include intraoperative neuromonitoring with cortical and subcortical mapping, awake brain surgery, fluorescent-guided resection with 5-aminolevulinic acid, and intraoperative imaging techniques such as ultrasound or MRI [36]. In case of eloquent located tumors, complete resection without causing a new permanent neurological deficit is not always feasible and residual tumor tissue can be left in situ as risk–benefit analysis [22, 28, 31, 37].

This risk–benefit analysis should always include a thoroughly information of the patient and his relatives. Possible increased survival times that can be achieved through an extreme aggressive resection in which permanent new neurological deficits are hazarded need to be traded off for the quality of life. Surgeons carefully need to discuss possible postoperatively affection of quality of life with patients and relatives: what does quality of life mean for the particular patient? Is there an adequate home care infrastructure and what kind of professional support will be needed? This decision-making process requires well-trained neurooncological surgeons and neurooncologists as well as a considerable information of patients and relatives. Additionally, apart from personal preferences and environment that might be willing to accept new neurological deficits post-op, one also needs to consider and clarify the impact from neurological deficits on survival [38]. Postoperative new neurological deficits can affect survival to the extent of abolishment of the benefit caused from complete resection.

### Impact factors on survival

Besides the molecular pattern, further variables are discussed to influence the course of the disease, explaining similar survival curves in the first months of follow up [8], and the existence of longtime survivors with MGMT negative GBM [39]. These variables are expected to be age at diagnosis, general condition, tumor location and pre- and post-op tumor volume [21, 40]. In accordance with that, in our study older patients and those with more residual tumor volume had a worse outcome. However, these findings were not statistically significant. With a postoperative median KPS of 90% patients of our study showed no difference between preoperative and postoperative scale. Patients were mostly operated under neuromonitoring and often awake surgery settings aiming to prevent new neurological deficits. We assume that operative procedure added to a favorable outcome but also to residual tumor voluminal as functional limits were achieved under surgery. The median post-op KPS in a comparable study, in which patients had a complete resection, was 90% as well [22]. In this study patients with mainly less frequent eloquent located lesions were enclosed which might have contributed to a comparable KPS under total resection.

### General limitations

Based on the strict inclusion criteria we analyzed data of a very homogenous group, which might also cause a selection bias. Due to the retrospective and monocentric study design, results may be less conclusive caused by small patient numbers especially in the subgroup analysis. However, here we present an analysis of residual tumor tissue throughout the whole course of disease, all patients were treated at the same neurosurgical department with the same adjuvant therapy scheme. Survival data of the other cohorts used for comparison between CR and residual tumor were extracted from literature. In one study, data was collected at the same hospital with comparable inclusion criteria, however data were not collected for this present study, particularly. Compared to other studies, a different cut-off to categorize MGMT promoter methylated and unmethylated glioblastomas might also have an impact [29]. Concerning evaluation of the clinical status measured by KPS in most of studies used for comparison, only little reference was made to the post-op KPS [21, 23]. Therefore, a comparison concerning the KPS as postoperative outcome score in patients with complete vs. incomplete resection between these study populations and the present reported data could not be made. In general, the KPS is only a restricted outcome measure as subtle cognitive impairment can hardly be measured with the KPS as it only measures the physical status but omits other parts that contribute to quality of life (e.g. spirituality). Therefore, additional neurocognitive and psychooncological assessments are crucial in neurooncological patients. Nevertheless, the KPS is an easily accessible and quickly performable tool and still important as a correlation of physical functioning deficits and significantly decreased quality of life has been reported. The KPS can assess the evolution of the clinical status of a glioblastoma patient and is therefore widely used in neurooncology.

At last**,** all histopathological diagnoses were re-classified according to the 2016 WHO guidelines, which lead to a loss of patients in which re-classification was not feasible. The risk of over- or underestimation of the manual tumor volume calculation was minimized since the same person calculated it in all cases.

## Conclusion

Our data underline the impact of the MGMT promoter methylation status in the treatment response of IDH-wildtype glioblastoma. Even after incomplete resection, patients with MGMT hypermethylated tumor demonstrated increased OS and PFS. However, our data revealed that the disadvantage of an incomplete resection cannot be outweighed by a favorable MGMT status in contrast to. Therefore, gross total resection of IDH-wildtype glioblastoma should remain the gold standard. Still, more studies about the behavior and treatment of residual tumor tissue are required. In times of personalized medicine and quality of life representing a major goal in oncology, neuro-oncologists need to inform the patients and their caregivers thoroughly about advantages and disadvantages of residual tumor volume versus possible neurological deficits in gross total resection especially in an eloquent tumor location.

## Data Availability

Original data are available on request.
